# The Effects of Enteral Nutrition in Critically Ill Patients with COVID-19: A Systematic Review and Meta-Analysis

**DOI:** 10.3390/nu14051120

**Published:** 2022-03-07

**Authors:** Omorogieva Ojo, Osarhumwese Osaretin Ojo, Qianqian Feng, Joshua Boateng, Xiaohua Wang, Joanne Brooke, Amanda Rodrigues Amorim Adegboye

**Affiliations:** 1 Faculty of Education, School of Health Sciences, Health and Human Sciences, University of Greenwich, Avery Hill Campus, Avery Hill Road, London SE9 2UG, UK; 2 Smoking Cessation Department, University Hospital, South London and Maudsley NHS Foundation Trust, Lewisham High Street, London SE13 6LH, UK; osarhumwese.ojo@slam.nhs.uk; 3 The School of Nursing, Soochow University, Suzhou 215006, China; 20195231027@stu.suda.edu.cn (Q.F.); wangxiaohua@suda.edu.cn (X.W.); 4 School of Science, University of Greenwich, Medway Campus, Central Ave, Gillingham, Chatham Maritime, Kent ME4 4TB, UK; j.s.boateng@greenwich.ac.uk; 5 Faculty of Health, Education and Life Sciences, Ravensbury House, Birmingham City University, City South Campus, Birmingham B15 3TN, UK; joanne.brooke@bcu.ac.uk; 6 Centre for Healthcare Research, Faculty of Health and Life Sciences, School of Nursing, Midwifery and Health, Coventry University, Priory Street, Coventry CV1 5FB, UK; ad6287@coventry.ac.uk

**Keywords:** COVID-19, enteral nutrition, early enteral nutrition, delayed enteral nutrition, parenteral nutrition, gastrointestinal intolerance, mortality, length of hospital stay, days on mechanical ventilation

## Abstract

Background: Patients who are critically ill with COVID-19 could have impaired nutrient absorption due to disruption of the normal intestinal mucosa. They are often in a state of high inflammation, increased stress and catabolism as well as a significant increase in energy and protein requirements. Therefore, timely enteral nutrition support and the provision of optimal nutrients are essential in preventing malnutrition in these patients. Aim: This review aims to evaluate the effects of enteral nutrition in critically ill patients with COVID-19. Method: This systematic review and meta-analysis was conducted based on the preferred reporting items for systematic review and meta-Analysis framework and PICO. Searches were conducted in databases, including EMBASE, Health Research databases and Google Scholar. Searches were conducted from database inception until 3 February 2022. The reference lists of articles were also searched for relevant articles. Results: Seven articles were included in the systematic review, and four articles were included in the meta-analysis. Two distinct areas were identified from the results of the systematic review and meta-analysis: the impact of enteral nutrition and gastrointestinal intolerance associated with enteral nutrition. The impact of enteral nutrition was further sub-divided into early enteral nutrition versus delayed enteral nutrition and enteral nutrition versus parenteral nutrition. The results of the meta-analysis of the effects of enteral nutrition in critically ill patients with COVID-19 showed that, overall, enteral nutrition was effective in significantly reducing the risk of mortality in these patients compared with the control with a risk ratio of 0.89 (95% CI, 0.79, 0.99, *p* = 0.04). Following sub-group analysis, the early enteral nutrition group also showed a significant reduction in the risk of mortality with a risk ratio of 0.89 (95% CI, 0.79, 1.00, *p* = 0.05). The Relative Risk Reduction (RRR) of mortality in patients with COVID-19 by early enteral nutrition was 11%. There was a significant reduction in the Sequential Organ Failure Assessment (SOFA) score in the early enteral nutrition group compared with the delayed enteral nutrition group. There was no significant difference between enteral nutrition and parenteral nutrition in relation to mortality (RR = 0.87; 95% CI, 0.59, 1.28, *p* = 0.48). Concerning the length of hospital stay, length of ICU stay and days on mechanical ventilation, while there were reductions in the number of days in the enteral nutrition group compared to the control (delayed enteral nutrition or parenteral nutrition), the differences were not significant (*p* > 0.05). Conclusion: The results showed that early enteral nutrition significantly (*p* < 0.05) reduced the risk of mortality among critically ill patients with COVID-19. However, early enteral nutrition or enteral nutrition did not significantly (*p* > 0.05) reduce the length of hospital stay, length of ICU stay and days on mechanical ventilation compared to delayed enteral nutrition or parenteral nutrition. More studies are needed to examine the effect of early enteral nutrition in patients with COVID-19.

## 1. Introduction

Enteral nutrition is the provision of enteral feed to patients who have functional guts but are unable to meet their nutritional requirements through the oral route alone [1,2]. Usually, these patients have neurological conditions, such as stroke, dementia, multiple sclerosis and motor neuron disease that affect their swallowing ability, whereas other patients may have intellectual disability or failure to thrive [1]. The use of enteral nutrition support in critically ill patients is also recommended [3].

Patients who are infected with coronavirus (SARS-CoV-2) (COVID-19) present with acute respiratory distress syndrome and may require urgent respiratory and hemodynamic support in the intensive care unit (ICU) [4]. It has been reported that 53% of elderly patients hospitalised with COVID-19 were malnourished [4,5]. According to Thibault et al. [4], nutritional support should be integrated into the global management of COVID-19.

A long stay in the ICU contributes to malnutrition, which could lead to the loss of skeletal muscle mass and function, poor quality of life, disability and morbidities that persist following discharge [6]. Comorbidities, such as diabetes, cardiovascular diseases and old age, are usually associated with high risk of malnutrition and poor outcomes in patients with COVID-19 [6]. In the presence of COVID-19 infection, reduced mobility, food intake and catabolic changes may predispose patients to malnutrition [6].

Over 93 million patients have been affected by coronavirus (COVID-19) caused by SARS-CoV-2 as of January 2021 [7]. Furthermore, there is evidence that about 30% of those hospitalised are admitted to ICU for ventilatory support [7]. According to the National Nurses Nutrition Group (NNNG) [8], wherever possible, oral nutrition support should be considered as first line nutritional intervention. However, for patients who may require mechanical ventilation, the use of clinically assisted nutrition and hydration would be essential to support patient recovery [9].

### 1.1. Description of the Intervention

Enteral nutrition may be delivered to intubated and ventilated patients with COVID-19 through different enteral feeding tubes based on the needs of the patient. Initially, patients who are infected with COVID-19, including those who develop ventilator-dependent chronic respiratory failure, may receive enteral nutrition by nasogastric tube (NGT) [10]. NGT is usually the preferred approach for patients requiring short term enteral nutrition support for about 2–3 weeks [10,11] and enables early commencement of enteral feeding [12].

The American Society of Parenteral and Enteral Nutrition (ASPEN) defined early enteral nutrition as enteral feeding that is commenced within 24 to 36 h of ICU admission or 12 h of intubation and placement of mechanical ventilation [13]. However, it is also useful to consider contraindications to NGT, such as the presence of oesophageal fistulae and obstruction of the oro-pharynx, which may affect the passage of the NGT [1]. Goyal et al. [10] also reported local complications, including nasopharyngeal/oropharyngeal erosions and oesophageal/gastric ulceration due to NGT placement.

Therefore, patients with COVID-19 requiring long term enteral nutrition support may be provided with a percutaneous endoscopic gastrostomy (PEG) tube [10]. According to Goyal et al. [10], enteral feeding can commence 4 h after PEG tube placement, usually with a low dose and then increasing to the full dose to meet the energy requirement of 15–20 kcal/kg of actual body weight.

In terms of the delivery of enteral feed, this can be through intermittent bolus feeding via enteral syringe and enteral feeding tube, gravity feed or the use of continuous enteral feeding pump. All these enteral feeding methods have their advantages and limitations. An enteral feeding pump should be used where possible in the acute setting, if the feed is to be delivered over a period of time, and the priority for pump allocation should be patients who are ventilated [8].

Therefore, the use of bolus feeding or gravity feeding should be considered in general ward settings where it is appropriate for patients and staff [8]. However, Martindale et al. [12] recommended the use of continuous instead of bolus enteral nutrition in critically ill patients with COVID-19 due to a significant reduction in the incidence of diarrhoea in continuous enteral feeding. Moreover, contact between the patients with COVID-19 and the healthcare team is reduced in continuous compared to bolus enteral feeding; thus, the risk of exposure to COVID-19 is also reduced [12].

### 1.2. Implementation of the Intervention

Patients who are critically ill with COVID-19 are usually in a state of high inflammation, increased stress and catabolism as well as increased energy and protein requirements [14,15]. Typical symptoms of COVID-19, such as coughing and breathlessness, dry mouth, loss of taste and smell and high body temperature, increase nutritional requirements, while the inflammatory response, which reduces appetite, may affect dietary intake [16]. Globally, COVID-19 patients have been reported to have reduced oral intake for 5 to 10 days before admission [15].

Therefore, it is useful to commence early enteral nutrition in these patients to maintain adequate nutritional status, gut barrier and immune function [2,14]. The use of enteral nutrition as the preferred route of managing intubated and ventilated ICU patients with COVID-19 has been recommended by The European Society for Clinical Nutrition and Metabolism (ESPEN) and American Society for Parenteral and Enteral Nutrition [2,6,13].

Medical nutrition therapy, including enteral nutrition, is one of the fundamental tools used to mitigate the challenges of COVID-19 infection [17]. Researchers reported that patients who were malnourished and with low immunity were at greater risk of mortality and poor prognosis [17]. There is evidence that early administration of enteral nutrition in these patients improves mortality and can reduce infection compared to patients with delayed or withheld enteral nutrition provision [12].

### 1.3. Why It Is Important to Do This Review

Despite the significant complications associated with COVID-19, it would appear that only limited attention has been given to nutrition therapy as part of the broader supportive care for the patients [18]. However, enteral nutrition provision presents its challenges, including the potential for adverse reactions, such as abdominal distention, diarrhoea and regurgitation [14].

For example, delay in the administration of enteral nutrition has been associated with gastrointestinal dysfunction and could impair overall nutritional status, while early commencement of enteral nutrition was associated with gastrointestinal intolerance in 30 to 70% of ICU patients [7]. However, the goal of enteral nutrition is to meet the energy and protein requirements of patients [19]. Therefore, the proper timing of enteral nutrition support and the provision of optimal nutrients are essential in preventing malnutrition in these patients [7]. To our knowledge, to date, no systemic review and meta-analysis has sought to assess the effect of enteral nutrition in patients with COVID-19.

### 1.4. Aim

This review aims to evaluate the effects of enteral nutrition in critically ill patients with COVID-19.

## 2. Method

This is a systematic review and meta-analysis, which was conducted based on the preferred reporting items for systematic review and meta-Analysis (PRISMA) framework [20].

### 2.1. Types of Studies

The studies included in this review were randomised controlled trials (RCTs), retrospective and prospective observational studies.

### 2.2. Types of Participants

Adult participants who were diagnosed with COVID-19 regardless of the existence of co-morbidities (e.g., diabetes) on enteral nutrition were selected for the review.

### 2.3. Types of Interventions

We compared critically ill patients with COVID-19 receiving early enteral nutrition with those having delayed enteral nutrition or parenteral nutrition, irrespective of the enteral feeding tube used for delivering the feed.

### 2.4. The Inclusion Criteria

Studies (randomised and observational) involving participants with COVID-19 and on enteral nutrition were selected for the review. In addition, only studies involving patients aged 18 years and over were included in this review.

### 2.5. The Exclusion Criteria

Studies excluded were those involving children with COVID-19, oral dietary intake and oral nutritional supplements. Furthermore, patients without COVID-19 were also excluded.

### 2.6. Types of Outcome Measures

The following was the primary outcome measure of interest:

- Mortality.

The secondary outcome measures included:

- Length of hospital stay (days).
- Length of ICU stay (days).
- Days on mechanical ventilation (days).
- The Sequential Organ Failure Assessment Score.

### 2.7. Search Methods for Identification of Studies

Health Sciences Research Databases (including MEDLINE, Academic Search Premier, APA PsycInfo, Psychology and Behavioral Sciences Collection, APA PsycArticles databases and CINAHL Plus with Full Text) were searched via EBSCO-host based on the population, intervention, control, outcome (PICO) framework (Table 1), from database inception until 3 February 2022. Furthermore, EMBASE database and Google Scholar were searched for relevant articles. The reference lists of articles were also searched. Searches were conducted using medical subject headings (MesH) and synonyms, and these were combined with Boolean operators (OR/AND).

The searches were conducted independently by two members of the research team (O.O. and O.O.O.) and cross-checked by other members of the team (X-H.W. J.B.). Duplicate results from the different databases were removed using EndNote (Analytics, Philadelphia, PA, USA).

### 2.8. Data Collection and Analysis

#### 2.8.1. Selection of Studies

**Figure 1:**
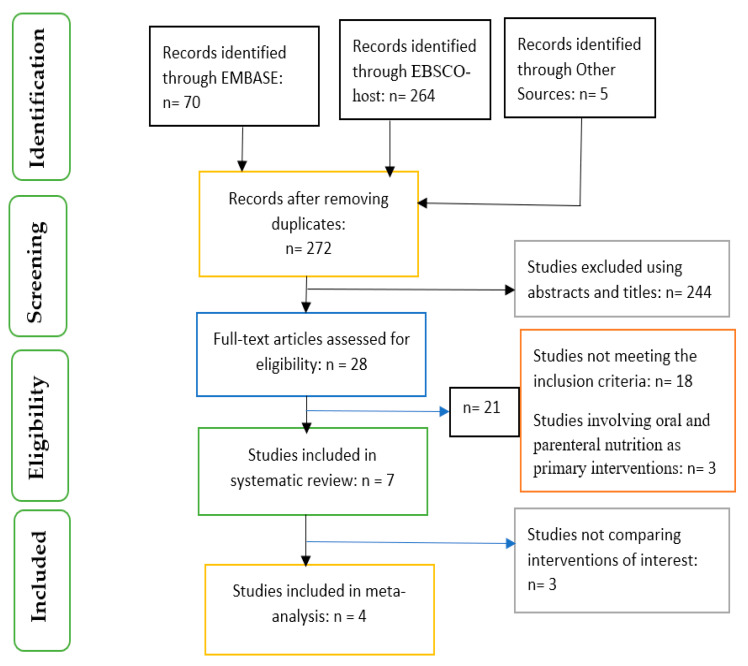
PRISMA flow chart on selection and inclusion of studies.

Based on the inclusion and exclusion criteria, a PRISMA flow chart (Figure 1) was used to select the articles included in the systematic review and meta-analysis.

#### 2.8.2. Data Extraction and Management

The information extracted from the studies included: country where the study was conducted, the type of study, sample size, mean age, study aim, type of intervention and main findings.

The data were extracted by one researcher (O.O.) from the articles included and crosschecked by two other members of the research group (O.O.O. and X-H.W.). The intervention group was compared with the control group based on the final values. With respect to studies that reported their findings as the median and first and third quartiles, the data were converted to means and standard deviations [21].

### 2.9. Data Analysis

Meta-analysis was conducted when there were enough studies reporting data on the same outcome. The Risk Ratio (RR) with 95% CIs was the statistical method used for the analysis of dichotomous data. On the other hand, continuous data were analysed as the mean difference (MD) with 95% CIs. The meta-analysis results are presented in the form of Forest plots, and *p* < 0.05 was used as the statistical significance of the overall effect of the intervention.

The I^2^ statistic expressed as a percentage was used to evaluate the level of heterogeneity of included studies [22]. A fixed-effects model was used for the meta-analysis for all the parameters of interest. On the other hand, a subgroup analysis was conducted for ‘Mortality’, which had sufficient studies included and to examine the effects of early versus delayed enteral nutrition and enteral nutrition versus parenteral nutrition on mortality in critically ill patients with COVID-19. The Review Manager (RevMan) 5.3 software [23] was used to conduct the meta-analysis.

## 3. Results

Seven studies were included in the systematic review, and four studies were used for the meta-analysis (Figure 1). Three studies were conducted in the US [24,25,26], and there was one study each from Brazil [27], Greece [28], Mexico [7] and China [29] (Table 2).

### 3.1. Assessment of Risk of Bias in Included Studies

**Figure 2:**
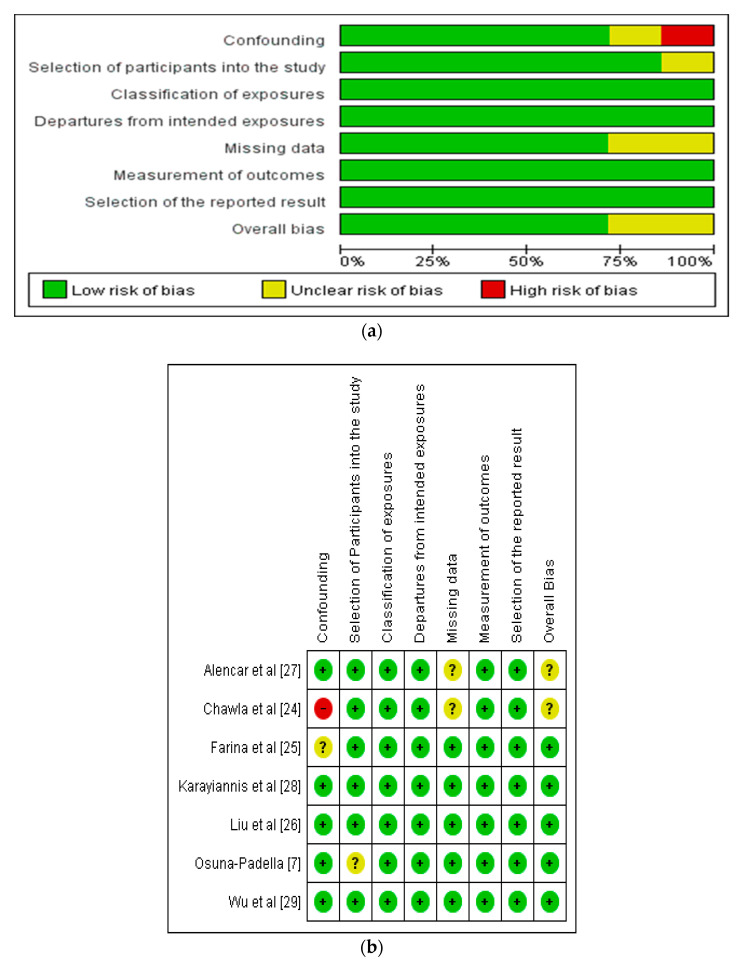
Represents (**a**) risk of bias graph and (**b**) risk of bias summary [7,24,25,26,27,28,29].

The risk of bias of included studies was evaluated by two researchers (Q-Q.F. and X-H.W.) using the Preliminary Tool for Risk of Bias in Exposure Studies [30]. The following domains were assessed; overall bias, selection of the reported result, measurement of the outcome, missing outcome data, departures from intended exposures, classification of exposures, selection of participants into the study and bias due to confounding variables (Figure 2a,b). All the studies demonstrated low risk of bias with respect to selection of the reported result, measurement of outcomes, departures from intended exposures and classification of exposures.

Two studies [24,27] showed an unclear risk of bias in relation to missing data, one study was with respect to the selection of participants [7], and another study was in relation to confounding variables [25]. Chawla et al. [24] showed a high risk of bias with respect to the confounding variables (refer to Figure 2).

Two distinct areas were identified from the results of the systematic review and meta-analysis: the impact of enteral nutrition and gastrointestinal intolerance associated with enteral nutrition. The impact of enteral nutrition was further sub-divided into early enteral nutrition versus delayed enteral nutrition and enteral nutrition versus parenteral nutrition.

### 3.2. The Impact of Enteral Nutrition

Alencar et al. [27] found that patients on an enteral nutrition diet of ≥25 kcal/kg of weight/day and ≥1.2 protein/kg of weight had a lower odds ratio in relation to mortality compared to patients who did not achieve these energy and protein levels. Furthermore, there were lower average maximum kcal/kg, protein/kg, percentage of diet adequacy and total caloric values among patients who died compared with patients who survived and were discharged [27]. According to Liu et al. [26], the median length of intensive care unit (ICU) stay of the 79 patients who did not receive enteral feeding was 2 days vs. 18 days for patients who received enteral tube feeding.

#### 3.2.1. Early Enteral Nutrition versus Delayed Enteral Nutrition

Chawla et al. [24] found that early enteral nutrition was associated with a lower hazard of in-hospital death and that the cumulative incidence of mortality was also lower in the early enteral nutrition group, with an estimated incidence of 40% and 53% at 10 and 20 days, respectively compared to 47% and 60% with delayed enteral nutrition. Chawla et al. [24] reported early enteral nutrition is useful in managing patients who are critically ill through the maintenance of gut integrity, stress modulation and attenuation of disease severity. Furthermore, the lower risk of in-hospital death in the early enteral nutrition group supports the need for this approach in patients with COVID-19 who are critically ill and intubated [24].

**Figure 3:**
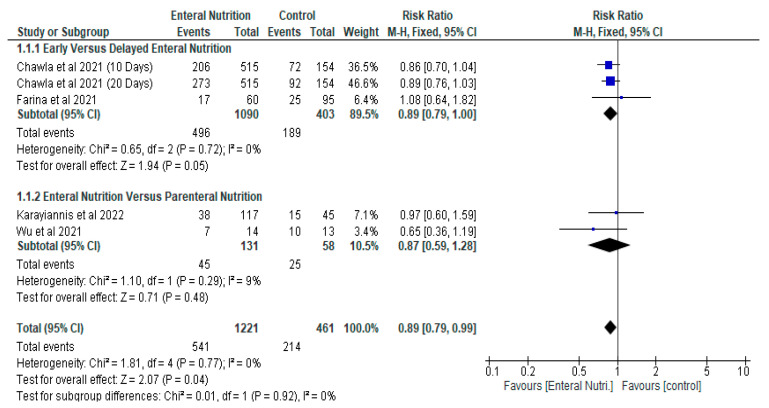
The effect of enteral nutrition on mortality.

In contrast, Farina et al. [25] reported the commencement of enteral nutrition within 24 h of starting mechanical ventilation may not improve outcomes in critically ill patients with COVID-19. The results of the meta-analysis of the effects of enteral nutrition in critically ill patients with COVID-19 showed that overall, enteral nutrition was effective in significantly reducing the risk of mortality in these patients compared with control, with a risk ratio of 0.89 (95% CI, 0.79, 0.99, *p* = 0.04) (Figure 3). Therefore, the Relative Risk Reduction (RRR) of mortality by enteral nutrition was 11%.

Four studies contributed to the analysis of mortality involving 1682 participants. Following sub-group analysis, the early enteral nutrition group (gp) also showed significant reduction in risk of mortality with a risk ratio of 0.89 (95% CI, 0.79, 1.00, *p* = 0.05) (Figure 3). Two studies [24,25] contributed to this analysis with 1493 participants (Early enteral nutrition gp, *n* = 1090. Delayed enteral nutrition gp, *n* = 403).

**Figure 4:**
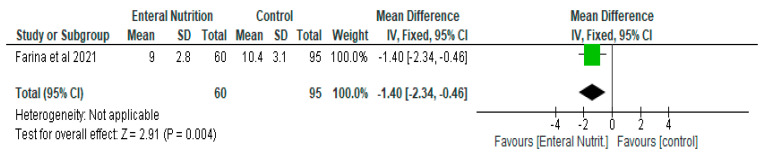
The effect of enteral nutrition on the Sequential Organ Failure Assessment score.

Only one study contributed to the results of the meta-analysis of the Sequential Organ Failure Assessment (SOFA) score [25]. There was a significant reduction in the SOFA score in the early enteral nutrition group compared with the delayed enteral nutrition group with a mean difference of −1.4 (95% CI, 2.34, −0.46, *p* = 0.004) (Figure 4).

#### 3.2.2. Enteral Nutrition versus Parenteral Nutrition

In the study conducted by Wu et al. [29], the levels of albumin were significantly higher in the enteral nutrition group compared with the parenteral nutrition group (*p* = 0.030) following 7 days of nutritional intervention. In addition, while the 28-day mortality was 50% in the enteral nutrition group, it was 76.9% in the parenteral nutrition group, and the Kaplan–Meier survival analysis showed profound differences between the two groups (*p* = 0.030) [29]. A total of 34.2% of the patients in the parenteral group had died at 30 days of hospitalization compared to 32.7% in the enteral group. The relative risk (RR) for the group receiving enteral nutrition was 0.97. Furthermore, patients with COVID-19 in the enteral group showed a lower duration of hospital stay [29].

**Figure 5:**
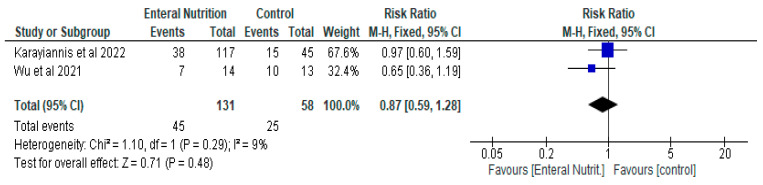
The effect of enteral nutrition compared to parenteral nutrition on mortality.

The result of the current meta-analysis, which compared enteral nutrition with parenteral nutrition in relation to mortality did not show any significant difference between the two groups with a risk ratio of 0.87 (95% CI, 0.59, 1.28, *p* = 0.48). Two studies [28,29] contributed to the analysis with 189 participants (Enteral nutrition gp, *n* = 131. Parenteral gp, *n* = 58) (Figure 5).

**Figure 6:**
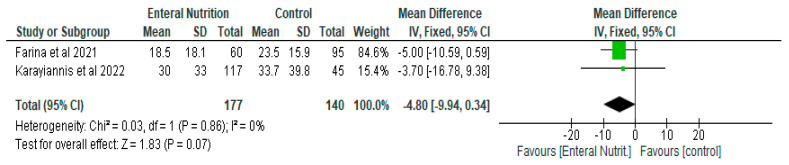
The effect of enteral nutrition on length of hospital stay (days).

**Figure 7:**
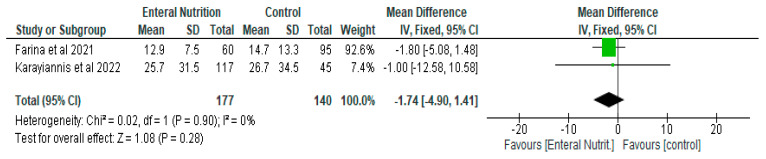
The effect of enteral nutrition on length of ICU stay.

**Figure 8:**
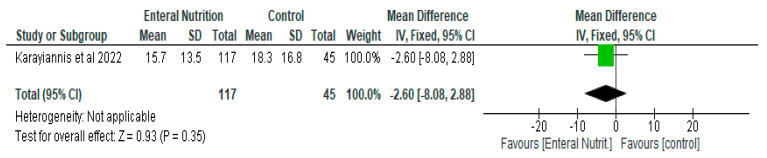
The effect of enteral nutrition on days on mechanical ventilation.

With respect to the length of hospital stay, length of ICU stay and days on mechanical ventilation, while there were reductions in the number of days in the enteral nutrition group compared to the control (delayed enteral nutrition or parenteral nutrition), the differences were not significant (Figure 6, Figure 7 and Figure 8). Enteral nutrition reduced the length of hospital stay with a mean difference of −4.80 (95% CI, −9.94, 0.34, *p* = 0.07) (Figure 6) and length of ICU stay with a mean difference of −1.74 (95% CI, −4.90, 1.41, *p* = 0.28) (Figure 7).

The mean difference between the enteral nutrition group and parenteral nutrition group with respect to days on mechanical ventilation was −2.6 (95% CI, −8.08, 2.88, *p* = 0.34) (Figure 8).

### 3.3. Gastrointestinal Intolerance Associated with Enteral Nutrition

Osuna-Padilla et al. [7] reported that gastrointestinal intolerance in the form of vomiting, diarrhoea and gastroparesis was present in 18 patients (35%) at any moment during the first 7 days of enteral nutrition. Furthermore, the incidence of constipation was found in 45 (87%) patients, while instability due to hemodynamics was the main reason (64%) for avoiding the provision of enteral nutrition in the first 24 h of mechanical ventilation [7].

Despite the gastrointestinal intolerance and interruptions, 90% of patients who received enteral nutrition had >80% of their nutritional requirements by day 7 (22.8 ± 7.3 kcal/kg) [7]. Therefore, Osuna-Padilla et al. [7] concluded that enteral nutrition was feasible and well-tolerated in critically ill patients with COVID-19 receiving invasive mechanical ventilation in the first week of intubation.

Similarly, Farina et al. [25] reported that the presence of gastrointestinal symptoms on admission did not appear to limit the ability to provide enteral nutrition for mechanically ventilated patients with COVID-19. However, Farina et al. [25] reported that feeding intolerance was a frequently encountered complication in critically ill patients with COVID-19 and that feeding intolerance was associated with poor outcomes, including higher rates of multiorgan dysfunction, longer ICU stays and increased mortality.

According to Liu et al. [26], 56% of the patients developed feeding intolerance, mostly in the form of large gastric residual volumes (83.9%), abdominal distension (67.2%) and vomiting (63.9%). Furthermore, patients with feeding intolerance were significantly more likely to suffer cardiac, renal, hepatic and hematologic complications during their hospitalization compared with feed-tolerant patients [26]. Karayiannis et al. [28] reported significant differences in relation to the rates of adverse gastrointestinal events between the parenteral group compared to the enteral group (32.3% vs. 22.1% for vomiting and 37.2% vs. 29.2% for diarrhoea, respectively with *p* < 0.05).

## 4. Discussion

The results of the systematic review and meta-analysis showed that early enteral nutrition was effective in significantly (*p* < 0.05) reducing the risk of mortality and SOFA score compared with delayed enteral nutrition in critically ill patients with COVID-19. The Relative Risk Reduction of mortality in patients with COVID-19 by early enteral nutrition was 11%. On the other hand, while early enteral nutrition or enteral nutrition may reduce the length of hospital stay, length of ICU stay and days on mechanical ventilation compared to delayed enteral nutrition or parenteral nutrition, respectively, these differences were not significant (*p* > 0.05).

The findings of this review agree with a previous meta-analysis of randomised controlled trials of early enteral nutrition given to patients within 24 h of ICU admission [31]. Tian et al. [31] found that mortality was reduced in the early enteral nutrition group compared with the delayed enteral group with an odds ratio of 0.45 (*p* = 0.038). Martindale et al. [12] further reported that there was evidence that early enteral nutrition improved mortality and reduced infections compared to delayed enteral nutrition or withheld enteral nutrition. Tian et al. [31] also found that there was no significant difference (*p* > 0.05) with respect to mortality between early enteral nutrition and parenteral nutrition.

According to Minnelli et al. [19], early enteral nutrition decreased mortality, length of ICU stay and risk of infection compared to delayed enteral nutrition in critically ill patients who did not have COVID-19. Therefore, the timing of nutrient delivery is crucial as enteral nutrition in ICU patients provides both macro and micronutrients, which help to to sustain gut integrity through the stimulation of blood flow in intraepithelial cells [19]. 

Yu et al. [32] compared the initiation time of enteral nutrition at the time of admission (intervention) to 24 to 48 h after admission (control) in patients who were critically ill and found that the serum albumin and pre-albumin were significantly higher (*p* < 0.05) in the intervention group compared with the control group (*p* < 0.05). Furthermore, the incidences of oesophageal reflux, pulmonary infection and gastric retention were significantly lower in the intervention group compared to the control group (*p* < 0.05) [32]. 

These factors may explain the differences (*p* < 0.05) between early enteral nutrition and delayed enteral nutrition and between enteral nutrition and parenteral nutrition in the current review, although the difference in the latter was not significant (*p* > 0.05). A number of international nutrition bodies and organisations, such as The American Society for Parenteral and Enteral Nutrition and the Society of Critical Care Medicine have recommended early enteral nutrition provision within 24–36 h of admission to the ICU or within 12 h of intubation in COVID-19 patients admitted in critical care units [12,13,19].

While it is recognised that COVID-19 is a respiratory disease, it has been shown that the disease can affect other organs of the body, including disrupting the normal intestinal mucosa of the digestive system, which could lead to a range of gastrointestinal symptoms and impaired nutrient absorption [2]. In addition, due to the poor appetite of some patients with COVID-19, including those who may be critically ill, it could be difficult for them to meet their daily nutritional requirements through the oral route alone.

These patients are at increased risk of nutritional deficit and should be screened for nutritional risk [14]. Therefore, the provision of nutrition support is essential in managing critically ill patients with COVID-19 [14]. In particular, the use of enteral nutrition may be the preferred route to enhance the integrity of the gut and promote immune function [2]. Vila and Gau [33] noted that early enteral nutrition is the best method of providing nutritional support in critically ill patients admitted to ICU.

Expert guidance recommends early feeding, usually within 24 to 48 h of ICU admission, including patients with COVID-19, preferably early enteral nutrition, which has been shown to decrease the incidence of nosocomial infections in these patients [33,34,35]. Enteral nutrition presents a lower infection risk and earlier gut function compared to parenteral nutrition in ICU patients [2,13].

Furthermore, there is evidence that parenteral nutrition can induce deleterious changes in physiology, although complications, such as diarrhoea, nausea, vomiting, aspiration and tube-related complications may also be present in patients on enteral nutrition [2]. For example, earlier gut function, fewer infectious complications, reduced length of stay, and reduced cost have been observed in enteral nutrition provision compared to parenteral [36]. Therefore, adequate monitoring of patients on enteral nutrition is an important strategy in reducing the risk of complications [2].

Early enteral nutrition has been associated with restored enterocyte mass and function in critically ill patients [37]. Furthermore, dysfunction of the organ and infectious complications may be reduced [37]. Patel et al. [37] noted that there was a trend towards greater change in 48-h SOFA score in patients who received early enteral nutrition, which is in line with the findings of the current review. However, the use of parenteral nutrition has been recommended if it is impossible to initiate early enteral nutrition [19].

### Limitation of the Review

Seven articles were included in the systematic review, while only four were included in the meta-analysis. The few articles included may limit the wider application of the findings of the review. One article was an accepted abstract.

## 5. Conclusions

The results of this review demonstrated that early enteral nutrition significantly (*p* < 0.05) reduced the risk of mortality among critically ill patients with COVID-19. However, early enteral nutrition or enteral nutrition did not significantly (*p* > 0.05) reduce the length of hospital stay, length of ICU stay and days on mechanical ventilation compared to delayed enteral nutrition or parenteral nutrition, respectively. More studies are needed to examine the effect of early enteral nutrition in patients with COVID-19.

## Figures and Tables

**Table 1:** Search terms and search strategy.

Patient/Population	Intervention	Comparator	Combining Search Terms
Patients with Corona-virus	Enteral nutrition	Control	
Patients with corona virus OR COVID-19 OR COVID-19 testing OR SARS-CoV-2	Nutrition, Enteral OR Enteral feeding OR Feeding, Enteral OR Tube feeding OR Feeding, Tube OR Gastric feeding tubes OR Feeding tube, Gastric OR Feeding tubes, Gastric OR Gastric feeding tube OR Tube, Gastric feeding OR Tubes, Gastric feeding		Column 1 AND Column 2

**Table 2:** General characteristics of included studies.

Citation/Country of Study	Type of Study	Sample Size	Mean Age (Years)	Aim	Interventions	Results
Alencar et al. [27] Brazil	Retrospective study	*n* = 112	<60 (*n* = 24) ≥60 (*n* = 88)	To evaluate the association between enteral nutrition support and clinical outcomes in patients with COVID-19	Enteral nutrition	The authors found an association between deficit in protein and energy supply and mortality and recommended that nutrition support should be promoted in such conditions.
Chawla et al. [24] USA	Retrospective study	*n* = 515	Not Available	To examine if early enteral nutrition reduced morbidity and mortality in patients with COVID-19	Early enteral nutrition versus delayed enteral nutrition	There was a lower risk of in-hospital death in the Early enteral nutrition group compared with the Delayed enteral nutrition group. The times to extubate and discharge patients from the hospital were not associated with early enteral nutrition administration.
Farina et al. [25] USA	Retrospective study	*n* = 155	60.3 ± 13.8	To assess the effect of early enteral nutrition on outcomes in mechanically ventilated patients with COVID-19	Early enteral nutrition versus delayed enteral nutrition	The commencement of early enteral nutrition within 24 h did not improve the outcomes in mechanically ventilated patients with COVID-19
Karayiannis et al. [28] Greece	Prospective observational study	*n* = 162	EN group (63.2 ± 11.9) PN group (62.7 ± 10.7)	To describe the feeding practices of intubated patients with COVID-19 and their association with mortality, length of hospital stay and mechanical ventilation.	Enteral nutrition compared with parenteral nutrition.	During the second week of ICU hospitalization, enteral feeding may be associated with a shorter duration of hospitalization and use of mechanical ventilation support in COVID-19 patients who are critically ill and intubated.
Liu et al. [26] USA	Retrospective study	Received enteral nutrition (*n* = 323) Did not receive enteral nutrition (*n* = 79)	59.6 ± 14.9 among 323 patients on enteral nutrition	To evaluate the prevalence and clinical outcomes of feeding intolerance among COVID-19 patients	Enteral nutrition versus ‘No enteral nutrition’	There were 56% incident cases of feeding intolerance among the 323 patients on enteral nutrition. The length of intubation, ICU admission and mortality were (16 versus 2 days), (18 versus 2 days) and (84 versus 18) among patients that received enteral tube feeding compared with no tube feeds, respectively.
Osuna-Padella et al. [7] Mexico	Retrospective study	(*n* = 52)	55.7 ± 14.3	To examine the incidence of gastrointestinal intolerance associated with enteral nutrition	Enteral nutrition during the first week of administration	Enteral nutrition was well tolerated in patients with COVID-19 within the first week of intubation and who are on mechanical ventilation.
Wu et al. [29] China	Retrospective study	enteral nutrition (*n* = 14) parental nutrition(*n* = 13)	74.9 ± 10.5	To evaluate the nutritional status of critically ill patients with COVID-19 and to determine, which route of nutrition support is advantageous	Enteral nutrition versus parenteral nutrition	The incidence of nutritional risk in critically ill patients with COVID-19 was very high. Early EN may be beneficial to patient outcomes.

Abbreviations: COVID-19 (coronavirus 2019); EN (enteral nutrition); and PN (parenteral nutrition).

## Data Availability

We conducted secondary data analysis of publicly available data.
